# Percutaneous Coronary Intervention Versus Robotic Coronary Bypass for Left Anterior Descending Artery Chronic Total Occlusion

**DOI:** 10.1016/j.jscai.2024.102278

**Published:** 2024-10-09

**Authors:** Elsa Hebbo, Wissam A. Jaber, Giancarlo Licitra, Bryan Kindya, Malika Elhage Hassan, Mariem Sawan, Nikoloz Shekiladze, Pratik B. Sandesara, William J. Nicholson, Michael E. Halkos

**Affiliations:** a Emory Clinical Cardiovascular Research Institute, Division of Cardiology, Department of Medicine, Emory University School of Medicine, Atlanta, Georgia; b Division of Cardiothoracic Surgery, Joseph B. Whitehead Department of Surgery, Emory University School of Medicine, Atlanta, Georgia

**Keywords:** chronic total occlusion, percutaneous coronary intervention, robotic-assisted bypass surgery

## Abstract

**Background:**

Both percutaneous coronary interventions (PCIs) and robotic-assisted coronary artery bypass (CAB) offer viable options for left anterior descending (LAD) chronic total occlusion (CTO) revascularization. Our study aims to compare long-term clinical outcomes associated with these 2 strategies.

**Methods:**

In this retrospective study, we analyzed data from 273 patients diagnosed with LAD CTO who underwent either PCI (n = 129) or CAB (n = 144) at a single institution. Long-term follow-up was available for 96 PCI and 125 CAB patients. We employed Kaplan-Meier curves and the log-rank test to conduct cumulative survival analyses free of major adverse cardiovascular events (MACE), cumulative survival, survival free of myocardial infarction, and repeat revascularization.

**Results:**

In the study cohort, patients who underwent PCI exhibited a higher prevalence of comorbidities including diabetes (48.9% vs 24.6%; *P* < .001), lower ejection fraction (44 ± 14 vs 52 ± 10; *P* < .001), prior heart failure (36.6% vs 22.2%; *P* = .02), and prior bypass surgery (16% vs 0, *P* < .001). PCI to non-LAD vessels was performed as part of initial complete revascularization in 40.3% of PCI and 40.6% of CAB patients. Upon a median 3.4 years of follow-up, CAB patients had significantly higher rates of survival free of MACE compared to PCI patients (unadjusted hazard ratio, 2.39; 95% CI, 1.13-5.03). Although PCI patients had similar unadjusted mortality, they experienced higher myocardial infarction and repeat revascularizations compared to CAB. However, the risk of repeat revascularization was attenuated after adjusting for prior bypass, diabetes, and ejection fraction.

**Conclusions:**

Among patients with LAD CTO, those undergoing robotic-assisted CAB had a higher 5-year overall survival free of MACE compared to those who underwent PCI. This discrepancy in outcomes can be attributed in part to the greater burden of comorbidities among PCI patients.

## Introduction

Chronic total occlusions (CTOs) pose a challenging category of obstructive coronary disease. Patients with CTO experience a notably worse overall prognosis with higher rates of deaths and major cardiovascular events.^1^ The principal indication to treat a CTO is symptom relief through optimal medical management or revascularization.^2^^,^^3^ The choice for revascularization includes percutaneous coronary intervention (PCI) or bypass surgery. The question of revascularization is specifically important and may be clinically prognostic for the left anterior descending (LAD) artery, which supplies a large myocardial territory and contributes to left ventricular function.^4^ The LAD is also unique in that its bypass can be achieved with a robotically-assisted left internal mammary artery (LIMA) harvesting and mini-thoracotomy, achieving off-pump arterial revascularization without the need for sternotomy.^5^ It is thought that a LIMA to the LAD has a long-term prognostic significance and may be an ideal revascularization mode for chronically occluded LAD.^6^

The recent advances in CTO PCI techniques have led to a significant improvement in the success rates of PCI to above 90%, with low complication and restenosis rates.^7^ A comparative assessment of PCI for LAD CTO vs coronary artery bypass (CAB) has not been explored. The present study aims to address this gap by comparing the long-term clinical outcomes between PCI and robotic-assisted CAB for LAD CTO.

## Methods

### Patient population

This retrospective cohort study compares patients who underwent either PCI or robotic-assisted CAB for LAD CTO at a single institution between 2010 and 2020. Baseline demographic, medical, angiographic, procedural, and follow-up data were collected from electronic health records. All procedures were performed by experienced operators. Patients with single and multivessel disease were eligible for inclusion, and management was not necessarily limited to the LAD, as other vessels in both groups may have undergone PCI as part of a complete revascularization approach. A follow-up questionnaire was used to assess for specific outcomes: death, myocardial infarction (MI), repeat revascularization, and stroke.

### Outcomes and study definitions

The primary outcome of the study was overall survival free of major adverse cardiovascular events (MACE), a composite of all-cause mortality, MI, and repeat revascularization over 5 years. The secondary outcomes were overall survival free of the components of MACE over 5 years.

Chronic total occlusion was defined as a 100% stenosis for a duration of at least 3 months. The coronary intervention was analyzed in the major epicardial arteries: left main, LAD, left circumflex artery, and right coronary artery. Coronary artery disease (CAD) in any vessel is defined as lesion >50% stenosis. Procedural success was defined as technical success without any in-hospital events. In-hospital events comprised death, MI, stroke, or urgent revascularization before discharge. Postprocedure complications included MI, stroke, death, new atrial fibrillation, pleural effusion, aortic dissection, pericardiocentesis, phrenic nerve injury, bleeding requiring transfusion of 2 or more red blood cell units, and renal failure. MI was defined according to the fourth universal definition of MI.^8^

### Hybrid revascularization

Coronary artery bypass was performed using the da Vinci robotic surgical platform (Intuitive Surgical) which allowed a sternal-sparing LIMA-LAD bypass. A camera and 2 instrument ports are introduced in the intercostal space at the midaxillary line. LIMA is harvested endoscopically. Next, a small thoracotomy incision is performed directly over the LAD, and on-pump LIMA to LAD anastomosis is performed. The revascularization of non-LAD vessels was performed using PCI, usually the day after the bypass. PCI is performed according to standard practices.

### Statistical analyses

Baseline characteristics were reported as percentages for categorical variables and means with standard deviations for continuous variables. Comparison between the 2 groups was analyzed with a χ^2^ test for categorical variables and an unpaired *t* test for continuous. *P* values < .05 are significant. Cumulative survival analyses, survival free of MI, repeat revascularization, and LAD revascularization were performed using Kaplan-Meier curves, and the difference between curves was assessed by log-rank test. Follow-up was truncated at 5 years and censored at the time of last contact. A Cox proportional hazard model was constructed to assess for confounders of the association between initial revascularization strategy and repeat revascularization. The presence of confounding was indicated if the adjusted estimate changed more than 10% from the crude model estimate. Statistical analyses were done using SPSS version 29.0.1.1 (IBM Corp). The research was carried out in accordance with the appropriate ethical guidelines and was approved by the Emory University Institutional Review Board.

## Results

### Study population and baseline

A total of 273 patients who underwent LAD CTO revascularization were included in this analysis. Baseline characteristics comparing PCI and CAB are summarized in Table 1. The average age of subjects was similar in both groups. For both strategies, the patients were predominantly male (81.4% vs 85.4%; *P* = .371) and White (71.7% vs 79.9%; *P* = .107). Both groups had similar percentages of active smokers (22.7% vs 21.5%; *P* = .40).

**Table 1:** Baseline characteristics.

Characteristics	CAB (n = 144)	PCI (n = 129)	*P* value
Age at procedure, y	62.11 ± 11.42	62.87 ± 10.64	.29
Male sex	123 (85.4%)	105 (81.4%)	.37
Race			.11
White/Caucasian	115 (79.9%)	81 (71.7%)	
Black/African American	20 (13.9%)	20 (17.7%)	
Other	9 (6.2%)	12 (9.3%)	
CAD presentation			<.001
Stable angina	46 (48.9%)	89 (69%)	
Unstable angina	31 (33%)	27 (20.9%)	
NSTEMI	4 (4.3%)	9 (7%)	
STEMI	1 (1.1%)	2 (1.6%)	
Other	12 (12.8%)	2 (1.6%)	
LVEF, %	52.82 ± 10.18	44.45 ± 14.33	<.001
Prior heart failure	33 (22.9%)	49 (38.3%)	.006
Coexisting atrial fibrillation	3 (2.1%)	12 (9.4%)	.008
Diabetes mellitus	39 (27.1%)	59 (45.7%)	.001
Dyslipidemia	142 (98.6%)	92 (71.3%)	<.001
Hypertension	130 (90.3%)	107 (82.9%)	.07
Actively smoking	31 (21.5%)	29 (22.7%)	.40
Prior myocardial infarction	64 (44.4%)	37 (31.6%)	.034
Prior valve surgery	0 (0%)	5 (4%)	.016
Prior PCI	58 (40.3%)	60 (46.9%)	.27
Prior CAB	0 (0%)	21 (16.3%)	<.001
Creatinine, mg/dL	1.09 ± 0.66 (144)	1.32 ± 1.37 (126)	.037
Currently on dialysis	2 (1.4%)	6 (4.7%)	.11
Peripheral arterial disease	14 (9.7%)	6 (4.7%)	.11
Chronic lung disease	21 (14.6%)	15 (11.6%)	.47
Cerebrovascular disease	11 (7.6%)	15 (11.6%)	.26
History of cancer	5 (3.9%)	4 (3.1%)	.73
Preprocedural CAD (stenosis > 50%)			
LAD CTO	144 (100%)	129 (100%)	–
LM	3 (25%)	9 (7.5%)	.14
LCX	34 (27.9%)	26 (20.2%)	.10
RCA	41 (33.6%)	46 (35.7%)	.42

Values are mean ± SD, n (%) or mean ± SD (n).

CAB, coronary artery bypass; CAD, coronary artery disease; LAD, left anterior descending; LCX, left circumflex; LM, left main, LVEF, left ventricular ejection fraction; NSTEMI, non-ST-elevation myocardial infarction; PCI, percutaneous interventions; RCA, right coronary artery; STEMI, ST-elevation myocardial infarction.

PCI patients exhibited a higher prevalence of comorbidities, including lower left ventricular ejection fraction (LVEF, 44 ± 14 vs 52 ± 10; *P* < .001), prior heart failure (36.6% vs 22.2%; *P* = .02), coexisting atrial fibrillation (9.4% vs 2.1%; *P* = .008), diabetes (48.9% vs 24.6%; *P* < .001), prior bypass surgery (16% vs 0%, *P* < .001), prior valve surgery (4% vs 0%; *P* = .016) and higher baseline creatinine (1.32± 1.37 vs 1.09 ± 0.66; *P* = .037). Conversely, the CAB group had significantly more patients with dyslipidemia (71.3% vs 98.6%; *P* < .001) and prior MI (31.6% vs 44.4%; *P* = .034) only. Patients in both groups had a similar distribution of vessel disease, as detailed in Table 1.

### Procedural characteristics

Procedural characteristics are described in Table 2. Procedural success rate was similar for both strategies (94.5% vs 94.4%; *P* = .48). PCI to non-LAD vessel was performed as part of a planned complete revascularization strategy in 40.3% of PCI and 40.6% of CAB patients. The revascularization of non-LAD vessels predominantly occurred simultaneously with the index procedure for PCI patients (55.8%) and postsurgery for CAB patients (64.9%). Notably, the right coronary artery stood out as the predominant nontarget vessel.

**Table 2:** Procedural characteristics.

Characteristics	CAB (n = 144)	PCI (n = 129)	*P* value
Procedural success^a^	94.4%	94.5%	.48
J-CTO score			
0	–	17 (14.7%)	–
1	–	39 (33.6%)	–
2	–	32 (27.6%)	–
3	–	25 (21.6%)	–
4	–	3 (2.6%)	–
Successful crossing strategy			
Antegrade wiring	–	79 (61.2%)	–
Antegrade dissection and reentry	–	20 (15.5%)	–
Retrograde	–	23 (17.8%)	–
None	–	7 (5.5%)	–
PCI to non-LAD	58 (40.6%)	52 (40.3%)	.97
Number of cases			
LAD into diagonal	15	6	–
RCA	29	34	–
LCX	29	20	–
Non-LAD PCI performed compared to LAD CAB or PCI			
Before	17 (29.8%)	14 (26.9%)	–
Same setting	3 (5.3%)	29 (55.8%)	–
After	37 (64.9%)	9 (17.3%)	–
Postprocedural complications^a^	44 (35.2%)	7 (7.3%)	<.001
Length of hospital stay, d	5.0 ± 2.5	1.5 ± 3.5	<.001

Values are n (%) or mean ± SD.

LAD, left anterior descending; LCX, left circumflex; PCI, percutaneous interventions; RCA, right coronary artery.

a Post procedure complications included myocardial infarction, stroke, death, new atrial fibrillation, pleural effusion, aortic dissection, pericardiocentesis, phrenic nerve injury, bleeding requiring transfusion of 2 or more red blood cell units, and renal failure.

Although none of the CAB patients had a previous bypass surgery, in the PCI group, 21 of 129 patients had a previously bypassed LAD, with 11 patients having an occluded or severely diseased bypass graft to the LAD. In the CAB group, 15 patients had concomitant LAD into diagonal stenting, all of which were simple nonbifurcation stenting. Conversely, in the PCI group, 33 of 129 patients (26%) had bifurcation stenting.

Significantly shorter hospital stays were observed for PCI patients compared to CAB patients (1.52 ± 3.52 vs 4.99 ± 2.51 days; *P* < .001). Additionally, postprocedural complications occurred less frequently in patients who underwent PCI (7.3% vs 35.2%; *P* < .001).

### Primary and secondary outcomes

**Figure 1:**
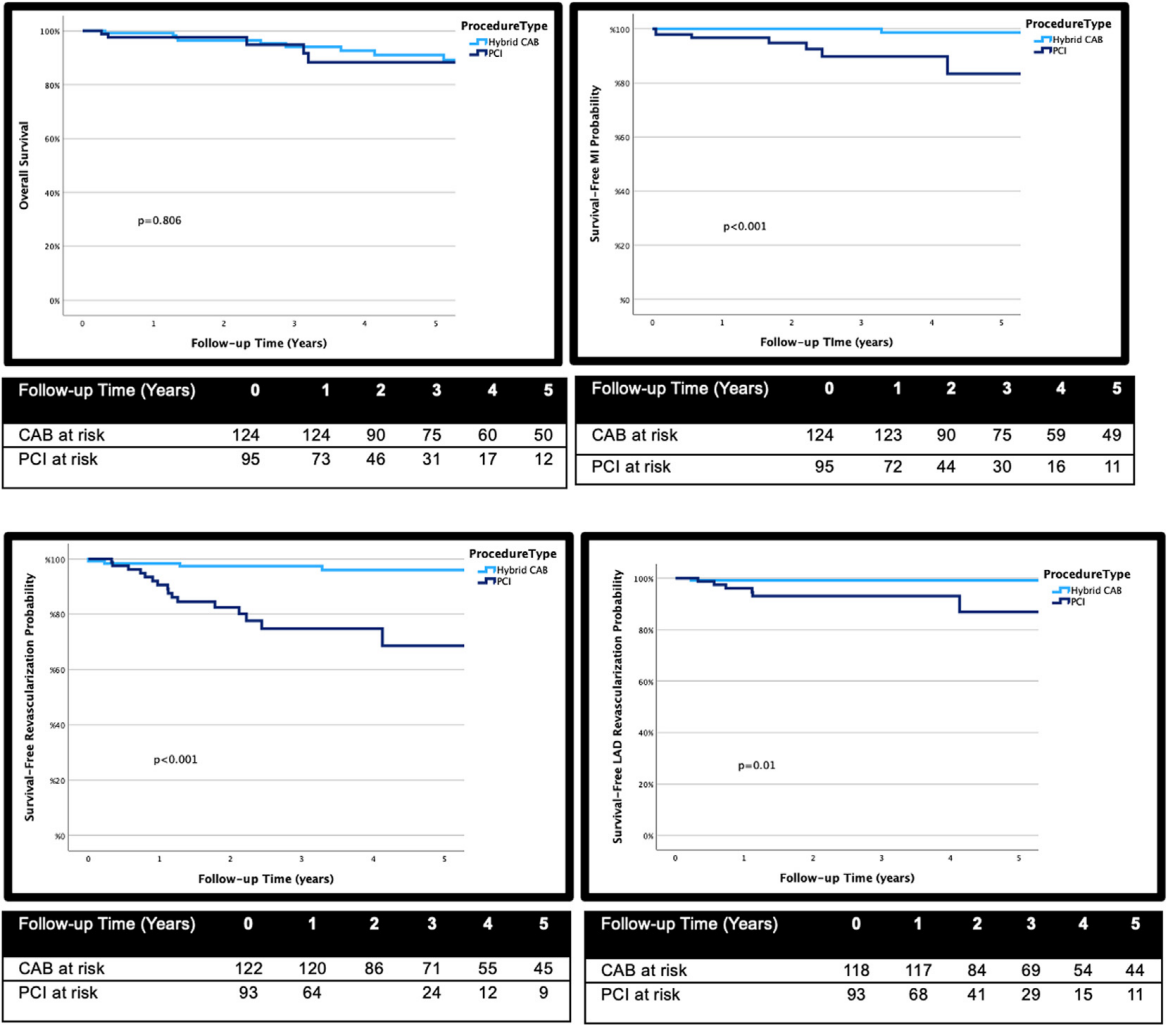
**Secondary outcomes.** Probability of overall survival, survival free of myocardial infarction (MI), survival free of repeat revascularization and survival free of left anterior descending (LAD) revascularization curves based on Kaplan-Meier estimates. CAB, coronary artery bypass; PCI, percutaneous coronary intervention.

Follow-up was successfully attained for 81% of patients, with a median follow-up duration of 3.4 years (Table 3). As depicted in the Central Illustration, the primary outcome of overall survival free of MACE significantly favored patients undergoing robotic CAB (*P* < .001). In terms of secondary end points, there was no significant difference in overall free survival between the 2 strategies (*P* = .806) (Figure 1). However, the rates of overall survival free of MI, repeat revascularization, and repeat LAD revascularization were significantly lower in PCI patients (*P* < .001, *P* < .001, and *P* = .01, respectively) (Figure 1). Repeat revascularization for the LAD was performed in 6 PCI patients and 2 CAB patients (6.3% vs 1.6%; *P* = .07).

**Table 3:** Outcomes at a median follow-up of 3.4 y.

Outcomes	CAB (125)	PCI (96)	*P* value
MACE	18 (14.4%)	24 (25%)	.05
Death	11 (8.8%)	7 (7.3%)	.307
MI	1 (0.8%)	7 (7.3%)	.01
Repeat revascularization	8 (6.4%)	3 (3.1%)	.005
CAB	1 (0.8%)	3 (3.1%)	.199
PCI	7 (5.6%)	16 (16.7%)	.008
Vessel undergoing repeat revascularization with PCI			
LAD	2	6	–
LCX	4	5	–
RCA	1	3	–

CAB, coronary artery bypass; LAD, left anterior descending; LCX, left circumflex; MACE, major adverse cardiovascular events; MI, myocardial infarction; PCI, percutaneous interventions; RCA, right coronary artery.

**Central Illustration:**
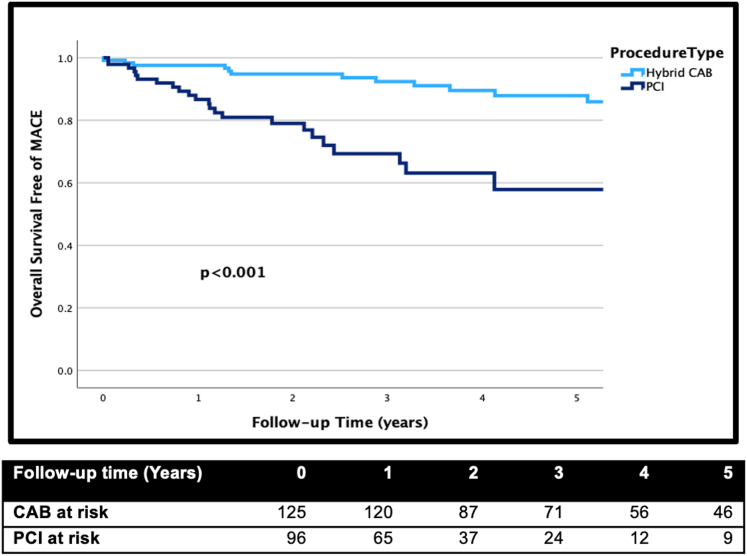
Overall survival free of major adverse cardiovascular events (MACE). CAB, coronary artery bypass; PCI, percutaneous coronary intervention.

An unadjusted Cox proportional model revealed a higher risk of repeat revascularization for PCI patients (HR, 7.0; 95% CI, 2.53-19.4). LVEF, diabetes, and prior bypass were found to be potential confounders. After adjusting for these factors, the association between PCI and repeat revascularization was attenuated (HR, 2.2; 95% CI, 0.6-7.6).

## Discussion

This study represents the largest comparison conducted within a single center, assessing both the immediate and long-term outcomes of PCI and robotic-assisted CAB in patients with LAD CTO. Our primary findings indicate that 5-year overall survival free of MACE favored CAB patients, primarily due to reduced rates of MI and repeat revascularization. The overall mortality was similar between the groups. Importantly, the PCI cohort had a higher burden of comorbidities; adjustment for baseline characteristics led to partial attenuation in the difference between the groups. Larger scale studies are needed to facilitate a more comprehensive comparison.

Several randomized controlled trials have examined the survival benefits of PCI for CTO. In the DECISION CTO trial, PCI significantly improved quality-of-life scores at 1 month, with sustained benefits observed up to 3 years compared to patients on optimal medical therapy.^9^^,^^10^ The Euro CTO trial demonstrated improvements in angina frequency, physical limitation, and quality-of-life in the PCI group at 1 year of follow-up.^10^^,^^11^ Similarly, a report from the OPEN CTO registry showed an improvement in health status after successful CTO PCI.^11^ However, when it comes to cardiovascular events, including death, MI, and repeat revascularization, these trials did not demonstrate additional benefits of PCI.^9^^,^^10^ Notably, these studies did not conduct analyses on a specific target vessel, such as the LAD.

In the EXPLORE trial, a subgroup analysis of LAD CTO showed improved LVEF at 4 months compared to patients who did not undergo PCI.^9^ Megaly et al^7^ report a 10.9% improvement in LVEF in patients with ischemic cardiomyopathy who underwent LAD CTO PCI. Although the debate on survival benefits of CTO PCI continues, the literature lacks comprehensive data comparing different revascularization methods for CTO.

Our study findings align with previous research on CTO revascularization. Strauss et al^12^ demonstrated that individuals undergoing revascularization via PCI or CAB had lower all-cause mortality compared to those without CTO revascularization. It also revealed that only revascularization through CAB significantly decreased repeat revascularization; nevertheless, it is essential to consider that patients in both groups had varying degrees of comorbidities.^13^ Prior CAB may reflect more aggressive atherosclerotic disease and complex comorbidities.^14^ The differences between the 2 groups could potentially be explained by the higher complexity of lesions in the PCI group. Native CAD progression is common post coronary artery bypass graft and studies have shown significant progression of proximal native vessel disease with increasing calcification.^15^ Additionally, post-coronary artery bypass graft CTO are typically more complex with higher J-CTO scores, and associated with lower technical success. Bifurcation LAD stenting was also common in the PCI group (26%), potentially adding to the increased risk of revascularization in this group.

An important finding in our study is that approximately 64.9% of patients undergoing robotic CAB for LAD CTO had PCI revascularization of non-LAD vessels. This integrates the 2 main revascularization modalities which is referred to as hybrid coronary revascularization.^16^^,^^17^ The major advantage of traditional CAB lies in the well-established durability of a LIMA-LAD graft. Substituting saphenous venous grafts with targeted PCI provides a reasonable alternative given that PCI with new drug-eluting stents might offer better efficacy than vein grafts.^17^

In our study, despite the fact that the majority of patients in the CAB group underwent hybrid revascularization, outcomes still favored CAB. This underscores the importance of the LIMA-LAD graft, especially in LAD CTO cases. The HYBRID trial and the Hybrid coronary REvascularization Versus Stenting or Surgery (HREVS) study have shown that compared to traditional CAB, hybrid CAB yields similar mortality and cardiovascular outcomes at 1 year.^18^^,^^19^ Consistent with our findings, the Hybrid coronary REvascularization Versus Stenting or Surgery trial indicated that patients with multivessel CAD who underwent PCI had similar mortality to the surgery cohort but an increased risk of repeat revascularization.^19^ However, these studies did not include patients with CTO.

Our group has previously published findings from a smaller cohort of patients with LAD CTO undergoing PCI or robotic CAB, revealing comparable short-term outcome.^20^ Building upon this, the current study includes a larger patient population and a longer follow-up period, uncovering disparities in MI and repeat revascularization rate between the 2 treatment groups.

Although hybrid revascularization presents an attractive approach for revascularizing multivessel coronary disease, especially in cases with LAD CTO, it is important to note that the patients in the current study were not randomized or matched, but rather specifically chosen by the treatment team for one revascularization mode or the other. Patients eligible for hybrid revascularization with CAB typically exhibit a good distal LAD target for robotic bypass, absence of severely reduced LVEF, absence of prior cardiac surgery, and non-LAD vessels that are amenable to PCI. Consequently, it becomes challenging to fully adjust for baseline differences when comparing CAB and PCI groups due to the inherent variability in patient selection for each strategy. Another limitation of the study is the incomplete follow-up in both groups, despite efforts to review charts and reach the patients. It is also important to note that many medical centers do not have the expertise to treat patients with either a robotic LIMA or with CTO PCI with the high success rates seen in this study, thus limiting the generalizability of the results.

## Conclusions

In conclusion, LAD CTO represents a unique category of CAD. Despite the abundance of studies done comparing revascularization methods, the focus on LAD CTO has been notably limited. Our study reveals that CAB offers superior 5-year overall survival free of MACE compared to PCI for patients with LAD CTO. Although the rates of all-cause mortality at 5 years were similar between the 2 cohorts, PCI patients had higher rates of MI and repeat revascularization, partly attributable to their higher burden of comorbidities.

## Declaration of competing interest

Wissam A. Jaber is a consultant, receiver of research grants, and educational grants from Medtronic, Abbott, Inari, Thrombolex. The other authors reported no financial interests.
